# Co‐delivery of dendritic cell vaccine and anti‐PD‐1 antibody with cryomicroneedles for combinational immunotherapy

**DOI:** 10.1002/btm2.10457

**Published:** 2022-11-27

**Authors:** Hao Chang, Xueyu Wen, Zhiming Li, Zhixin Ling, Yanting Zheng, Chenjie Xu

**Affiliations:** ^1^ The Cancer Hospital of the University of Chinese Academy of Sciences (Zhejiang Cancer Hospital) Institute of Basic Medicine and Cancer (IBMC), Chinese Academy of Sciences Hangzhou Zhejiang China; ^2^ Department of Biomedical Engineering City University of Hong Kong Hong Kong China; ^3^ College of Pharmaceutical Science, Zhejiang University of Technology Hangzhou Zhejiang China

**Keywords:** anti‐PD‐1, cancer immunotherapy, cryomicroneedles, dendritic cells, transdermal delivery

## Abstract

Combinational immunotherapy of dendritic cell (DC) vaccines and anti‐programmed cell death protein 1 antibodies (aPD1) has been regarded as a promising strategy for cancer treatment because it not only induces tumor‐specific T cell immune responses, but also prevents failure of T cell functions by the immune suppressive milieu of tumors. Microneedles have emerged as an innovative platform for efficient transdermal immunotherapies. However, co‐delivery of DC vaccines and aPD1 via microneedles has not been studied since conventional microneedle platforms are unsuitable for fragile therapeutics like living cells and antibodies. This study employs our newly invented cryomicroneedles (cryoMNs) to co‐deliver DC vaccines and aPD1 for the combinational immunotherapy. CryoMNs are fabricated by stepwise cryogenic micromoulding of cryogenic medium with pre‐suspended DCs and aPD1, which are further integrated with a homemade handle for convenient application. The viability of DCs in cryoMNs remains above 85%. CryoMNs are mechanically strong enough to insert into porcine and mouse skin, successfully releasing DCs and aPD1 inside skin tissue after melting. Co‐delivery of ovalbumin (OVA)‐pulsed DCs (OVA‐DCs) and aPD1 via cryoMNs induced higher antigen‐specific cellular immune responses compared with the mono‐delivery of OVA‐DCs or aPD1. Finally, administration with cryoMNs co‐encapsulated with OVA‐DCs and aPD1 increases the infiltration of effector T cells in the tumor, resulting in stronger anti‐tumor therapeutic efficacy in both prophylactic and therapeutic melanoma models compared with administration with cryoMNs loaded with OVA‐DCs or aPD1. This study demonstrates the great potential of cryoMNs as a co‐delivery system of therapeutic cells and biomacromolecules for combinational therapies.

## 1 INTRODUCTION

Dendritic cells (DCs) are professional antigen‐presenting cells and capable of activating antigen‐specific T cells that can recognize and eliminate tumor cells.^1^ Although DC vaccines have been demonstrated high safety and immunogenicity, clinical trials showed limited clinical efficacy of DC vaccination.^2^, ^3^ One reason is the immunosuppressive microenvironment of tumors that weakens the infiltration and effect of activated T cells.^4^ An important underlying mechanism of immunosuppression is the programmed cell death protein 1 (PD‐1) pathway. Interaction of PD‐1 receptors expressed on T cells and PD‐1 ligands (PD‐L1 and PD‐L2) expressed on tumor cells can induce T cell apoptosis, anergy, and exhaustion, which results in so‐called immune escape or immune tolerance.^5^ Immune checkpoint blockade (ICB) therapy, namely, blocking this pathway by using anti‐PD‐1 monoclonal antibodies (aPD1), has shown great promises at the restoration of T cell function.^6^ Therefore, DC vaccination combined with aPD1 is regarded as an appealing strategy for cancer immunotherapy, where DCs can foster activation of initial antigen‐specific T cells and aPD1 can subsequently maintain antitumor functions of T cells.^7^, ^8^

Microneedle (MN) patch is an array of micrometer‐sized needles. It has been extensively developed for the delivery of drugs in an easy‐to‐use, minimally invasive, effective and patient‐friendly manner.^9^, ^10^, ^11^ Due to the presence of a large population of immune cells in the dermal layers of skin, MNs have been demonstrated to be an effective vaccination strategy because they can enhance immunogenicity.^12^, ^13^, ^14^, ^15^ Recently, MNs are attracting a lot of interests for localized delivery of immune checkpoint inhibitors such as aPD1/PD‐L1, which are demonstrated to increase the therapeutic efficacy of ICB and minimize the undesirable side effects.^16^, ^17^, ^18^, ^19^, ^20^ Different types of MNs have been developed for encapsulating the aPD1/PD‐L1 for either monotherapy or combinational therapy. For example, Wang et al. introduced hyaluronic acid MNs integrated with pH‐sensitive dextran nanoparticles (NPs) that encapsulate aPD1 and glucose oxidase (GOx), which achieved the stimuli‐responsive release of aPD1 in tumor environment.^19^ Lan et al. developed a MN patch loaded with pH‐responsive tumor‐targeted lipid nanoparticles to co‐encapsulate aPD1 and cisplatin for synergistic cancer immune‐chemotherapy.^17^ Despite the achievements, co‐delivery of DC vaccines and aPD1 via MNs to synergize therapeutic action has not been studied yet. Since DC vaccines belong to living cell therapeutics, existing MN platforms are unsuitable for encapsulation and delivery of DC vaccines.

We recently introduced a new MN technology named cryomicroneedles (cryoMNs) that permits encapsulation and intradermal delivery of living mammalian cells.^21^ As a proof of concept, cryoMNs loaded ovalbumin‐pulsed DCs (OVA‐DCs) were used to immunize mice as prophylactic cancer vaccines, which significantly lowered the tumor burden compared to conventional intravenous and subcutaneous injections of OVA‐DCs. In this study, we employed the cryoMNs as the co‐delivery system of OVA‐DCs and aPD1 and investigated their combinational therapeutic efficacy in cancer immunotherapy. While the optimized formulation of the cryogenic medium was adopted from the previous report to fabricate cryoMNs, this study combined cryoMNs with a 3D‐printed handle that could greatly facilitate the practical usage of cryoMNs. CryoMNs could maintain the viability of DCs and are mechanically strong enough to penetrate the skin, successfully releasing the loaded DC and aPD1 inside skin tissue. We demonstrated that co‐delivery of OVA‐DCs and aPD1 via cryoMNs elicited higher antigen‐specific cellular immune responses and led to better anti‐tumor therapeutic efficacy in both prophylactic and therapeutic melanoma models compared with mono‐delivery of either OVA‐DCs or aPD1.

## 2 RESULTS AND DISCUSSION

### 2.1 Fabrication and characterization of cryoMNs co‐encapsulated with antigen‐pulsed DCs and aPD‐1

**FIGURE 1:**
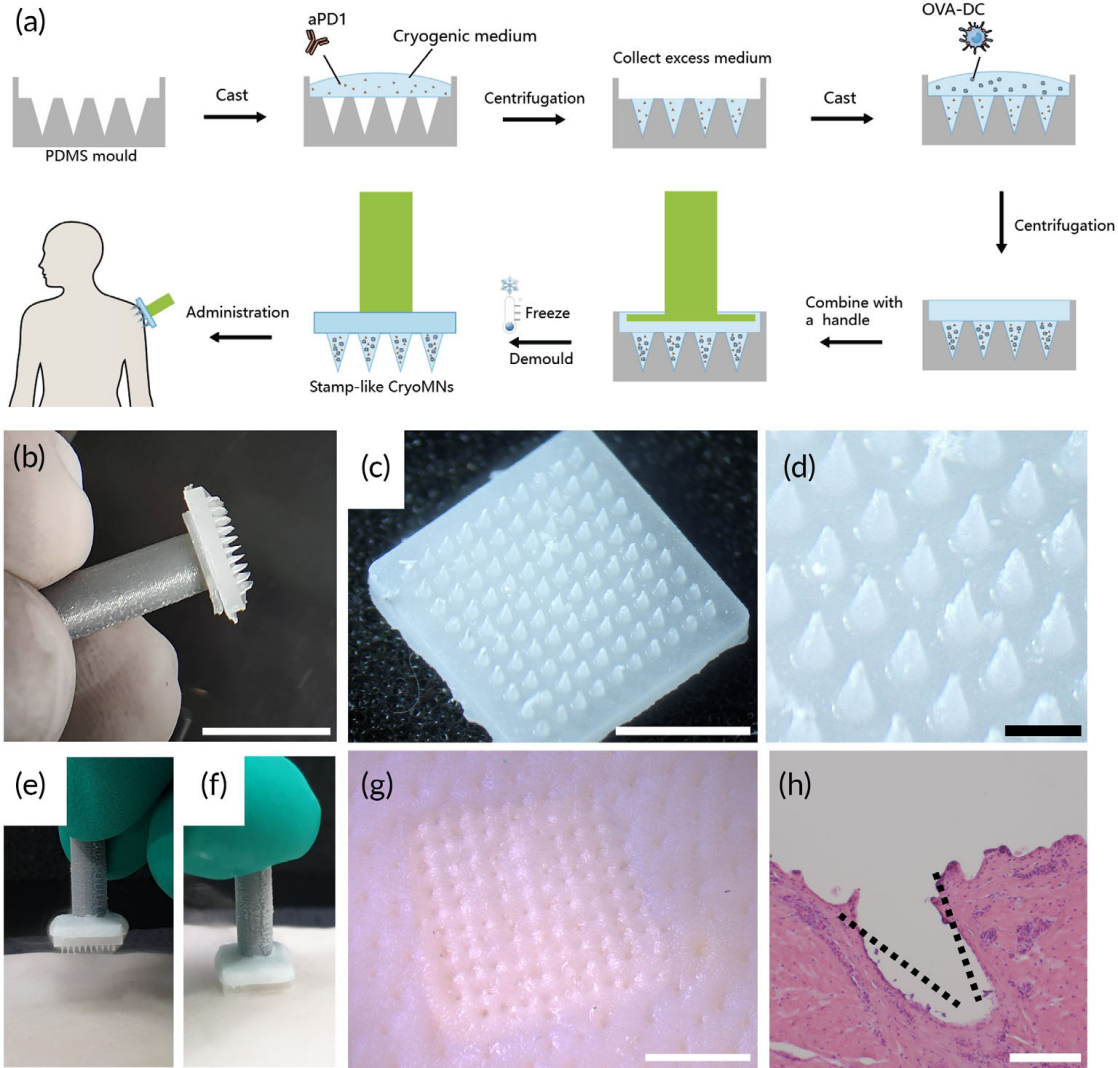
Fabrication and characterization of cryoMNs. (a) Schematic of the procedure to fabricate cryoMNs. (b) Photo of the cryoMNs combined with a handle that can be easily held. Scale bar: 10 mm. (c) Morphology of cryoMNs. Scale bar: 5 mm. (d) Magnified image of cryoMNs. Scale bar: 1 mm. (e) Before and (f) during administration of cryoMNs on the porcine skin. (g) Photo of the porcine skin after CryoMN administration. Scale bar: 5 mm. (h) Representative histological image of porcine skin inserted by cryoMNs. Scale bar: 500 μm

The cryoMNs were fabricated by the stepwise cryogenic micromoulding method as shown in Figure 1a. The optimized cryogenic medium, namely, phosphate‐buffered saline (PBS) supplemented with 2.5% (v/v) DMSO and 100 mM sucrose, was adopted from the previous report to fabricate cryoMNs.^21^ By using this formulation, cryoMNs can be successfully peeled out from the mold and keep the maximum viability of load cells.^21^ CryoMNs have to be used immediately after removal from their cryopreservation environment (−196°C, liquid nitrogen) to guarantee effective skin penetration. In the practical application, any touch with user's fingers or hand would cause the melt of cryoMNs due to the body temperature, making it challenging to use. Inspired by the handle stick of ice cream, we introduced a homemade handle by 3D printing and further combined it with cryoMNs during the fabrication process (Figure 1a). In this manner, the cryoMNs could be easily held with the user's finger without touching the main body of cryoMNs, which would greatly facilitate the usage of cryoMNs (Figure 1b). The fabricated cryoMNs had a height of ~950 μm and a base width of ~400 μm (Figure 1c,d). The conical structure and sharpness of each MN tip were well replicated despite the dimensional difference between the original template and cryoMNs due to the shrinking of the elastic PDMS during templating.

Next, the actual skin insertion capability of cryoMNs was examined with fresh porcine cadaver skin which is structurally similar to human skin.^22^ CryoMNs were inserted into the porcine skin with the assistance of the handle after being removed from the liquid nitrogen (Figure 1e,f). The microchannels created by cryoMNs were clearly observed immediately after application (Figure 1g). Histological analysis revealed that the efficient penetration depth of MN was about 400 μm (Figure 1h), suggesting the cryoMNs were strong enough to penetrate the skin.

### 2.2 Viability of DCs loaded in cryoMNs

**FIGURE 2:**
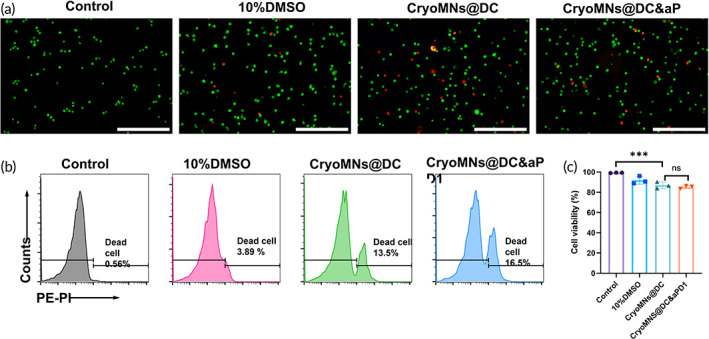
Viability of DCs released from cryoMNs. (a) Living (Green)/Dead (red) staining images of DCs. Control: Living DCs without any treatment. 10%DMSO: DCs were frozen in traditional cryopreservation formulation, namely, normal DC culture medium supplemented with 10%(v/v) DMSO. Scale bar: 200 μm. (b) Representative flow cytometry images of DCs stained with propidium iodide (PI). (c) Quantification of the percentage of living DCs in different groups. The data were calculated from flow cytometry results. Data are presented as mean ± SD (*n* = 3). ***p < 0.001; ns, *p* > 0.05, no significant difference

The total dosage of DC and aPD1 in one patch were 1 × 10^5^ cells and 6 μg, respectively. The cryoMNs loaded with DCs or aPD1 alone, and co‐encapsulated with DCs and aPD1 were named hereafter CryoMNs@DC, CyoMNs@aPD1 and CryoMNs@DC&aPD1, respectively. The viability of DCs loaded in cryoMNs was evaluated. Cell viability was tested after melting prepared cryoMNs in PBS (37°C). As shown in Figure 2a, most DCs released from both CryoMNs@DC and CryoMNs@DC&aPD1 were labeled with calcein AM (indicating live cells, green) and showed very little fluorescence signals of propidium iodide (PI, indicating dead cells, red). Furthermore, we used flow cytometry to quantify the percentage of living DCs. The viability of DCs from CryoMNs@DC and CryoMNs@DC&aPD1 was 86.9 ± 3.3% and 85.1 ± 1.4%, respectively (Figure 2b,c), which indicates the involvement of aPD1 in cryoMNs did not influence the viability of DCs. It could be expected that the cryoMNs are of great potential as a co‐delivery system of therapeutic cells and biomacromolecules for combinational therapies. However, for different combinations of therapeutic cells and biomacromolecules, it is still worth searching optimal formulation for fabricating cryoMNs in order to maximize the stability of cryoMNs and their therapeutic cargos.

### 2.3 Intradermal co‐delivery of DC and aPD1 with cryoMNs ex vivo and in vivo

**FIGURE 3:**
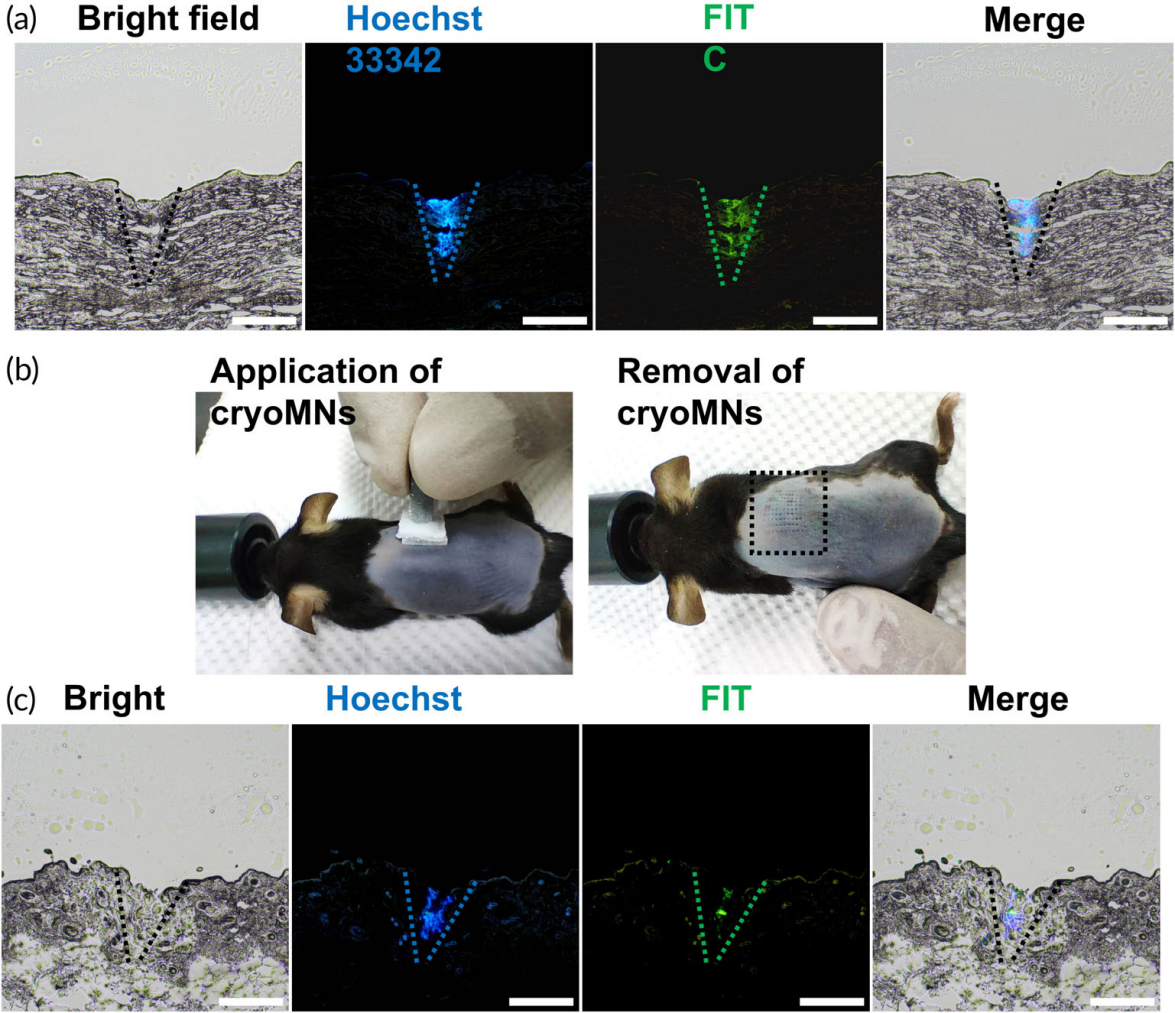
Skin insertion of cryoMNs co‐encapsulated with DCs and aPD1. (a) Fluorescent images of porcine skin cryosection after application of cryoMNs loaded with DCs and FITC‐aPD1. The DC nucleus was stained with NucBlue Live ReadyProbes reagent in advance. The needle shape is outlined with dashed lines. Scale bar, 200 μm. (b) Photographs of the mouse skin during and after application of cryoMNs with handle. The injection site is bounded by the black dotted square. (c) Fluorescent images of mouse skin cryosection after application of cryoMNs loaded with DCs and FITC‐aPD1. Scale bar, 200 μm

To facilitate the visualization of the DCs and aPD1 in skin, DCs were stained with Hoechst 33342 and FITC conjugated aPD1 (FITC‐aPD1) was used to package inside cryoMNs. Fluorescence signals of nuclear staining (blue) and FITC (green) were visualized inside porcine tissue (Figure 3a), which confirms the successful intradermal delivery of DCs and aPD1 with cryoMNs. Furthermore, the same cryoMNs were applied to the mouse dorsal skin with assistance of the handle (Figure 3b). After removal of cryoMNs, the microholes were clearly observed. Fluorescent imaging of the skin cryosections (Figure 3c **)** revealed that DCs and aPD1 were delivered in the dermis with depths ranging from about 20 to 200 μm (the thickness of the mouse epidermis is ~15 μm). Skin has been regarded as a highly immunoreactive site because it contains multiple immune cells and rich lymphatic vessels in the dermis layer.^23^, ^24^ This intradermal delivery of DCs and aPD1 is a prerequisite for inducing an effective immune response. In the hospital setting, intradermal injection is traditionally performed manually by the Mantoux's technique, which is technically challenging and requires skilled medical personnel.^25^ Due to the innate features of microdimensions, cryoMNs greatly simplifies the operation of intradermal injection.

### 2.4 Co‐delivery of OVA‐DCs and aPD1 with cryoMNs enhances antigen‐specific immune responses in vivo

**FIGURE 4:**
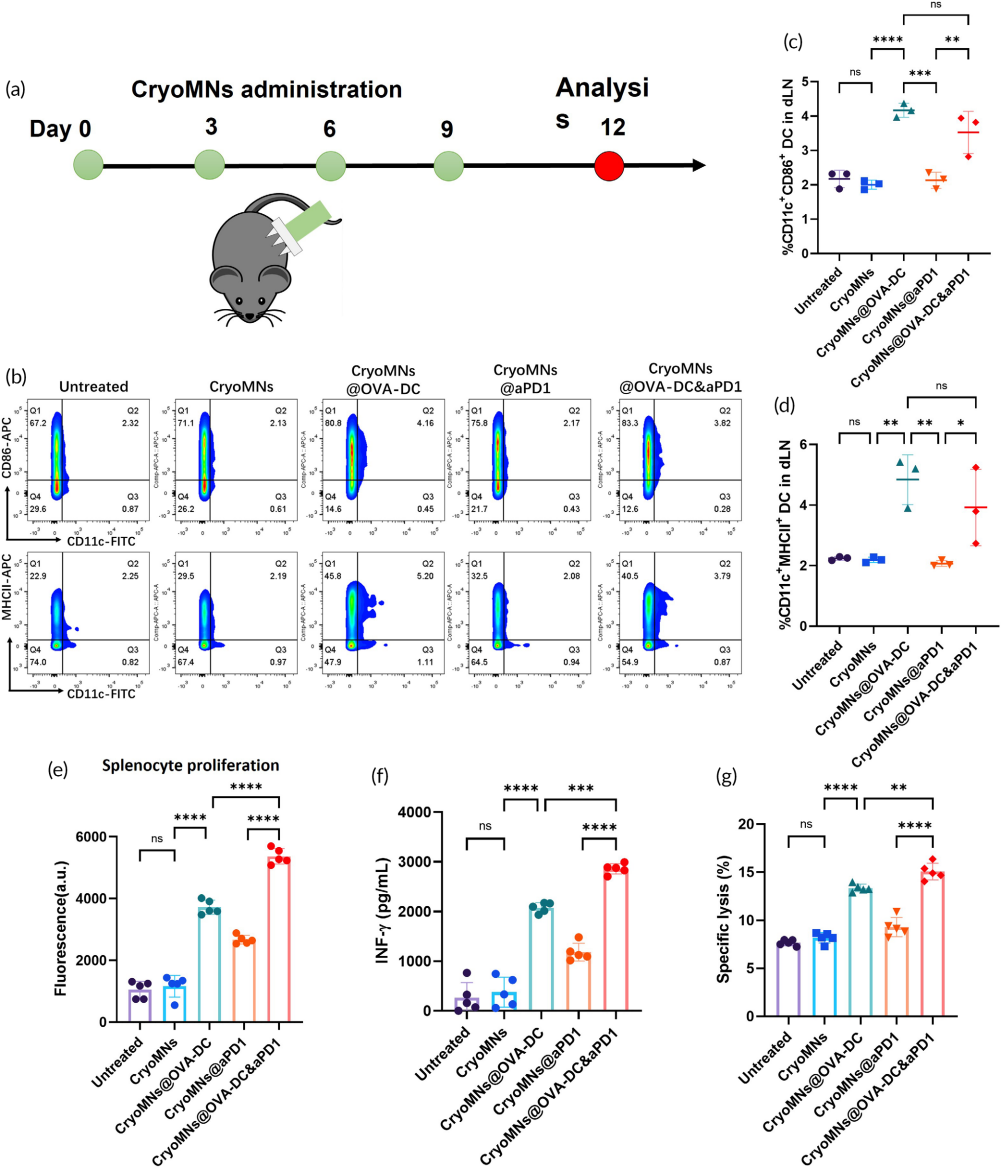
In vivo immuno responses after administration with cryoMNs. (a) Schematic of administration of cryoMNs. (b) Representative flow cytometry plots of CD11c^+^CD86^+^ DCs and CD11c^+^MHCII^+^ DCs in draining lymph nodes (dLNs) excised from mice in different treatment groups. Quantification of percentage of CD11c^+^CD86^+^ DCs (c) and CD11c^+^MHCII^+^ DCs (d) in dLNs. Data are presented as mean ± SD (*n* = 5). (e) In vitro proliferation of extracted splenocytes restimulated with 50 μg/ml antigen (OVA). The fluorescence intensity was obtained using an alamarBlue assay. (f) Level of IFN‐γ secreted by splenocytes re‐stimulated with 50 μg/ml OVA after 48 h of culture. Data are presented as mean ± SD (*n* = 5). (g) Determination of cytotoxic T lymphocyte (CTL) activity in vitro. Effector cells (splenocytes) and target cells (B16‐OVA cells) were cocultured at ratio of 100: 1, the lactate dehydrogenase (LDH) levels in the culture supernatants were measured. Data are presented as mean ± SD (*n* = 5). ns, *p* > 0.05, no significant difference; *p < 0.05; **p < 0.01; ***p < 0.001; ****p < 0.0001

Next, mice received administration of blank cryoMNs, CryoMNs@OVA‐DC, CryoMNs@aPD1, and CryoMNs@OVA‐DC&aPD1 according to the designated schedule and immune responses to different treatments were evaluated (Figure 4a). On day 12, activation and maturation of DCs in draining lymph nodes (dLNs) were examined by detecting the expression of major histocompatibility complex class II (MHCII) and the costimulatory molecule CD86 (Figure 4b–f). Administration of CryoMNs@OVA‐DC&aPD1 induced 3.52 ± 0.62% CD11c^+^CD86^+^ DCs and 3.92 ± 1.26% CD11c^+^MHCII^+^ DCs in the dLNs. These values were similar to administration of CryoMNs@OVA‐DC (4.16 ± 0.20% and 4.84 ± 0.82%, respectively), but significantly higher compared with administration of CryoMNs@aPD1 (2.13 ± 0.24% and 2.17 ± 0.09%, respectively) (Figure 4b–d). In addition, there is no significant difference in the percentage of CD11c^+^CD86^+^ DCs and CD11c^+^MHCII^+^ DCs between CryoMNs@aPD1 treated group and control groups (Untreated and CryoMNs treated group) (Figure 4c,d). These results suggest that administration of OVA‐DC is the key factor in homing of activated and mature DCs to lymph nodes, which will be important for the following antigen presentation between DCs and T cells to induce adaptive immune responses.^26^

After 2 days of culture, splenocytes from the mice administrated with CryoMNs@OVA‐DC&aPD1 exhibited a much more obvious proliferation response and secreted a higher level of interferon‐γ (IFN‐γ) after OVA restimulation, compared with those from mice administrated with CryoMNs@OVA‐DC and CryoMNs@aPD1. Cytotoxic T lymphocytes (CTL) play an important role in the inhibition of infection and tumor growth.^27^, ^28^ To confirm whether the co‐delivery of OVA‐DC and aPD1 can induce OVA‐specific CTLs in vivo, the splenocytes (effector cells) from different treated mice were incubated with B16 melanoma cell line transfected with ovalbumin (B16‐OVA, targeted cells). CTL‐mediated cytotoxicity was measured using LDH release assay. As depicted in Figure 4g, splenic T lymphocytes from mice administrated with CryoMNs@OVA‐DCs&aPD1 lysed greater numbers of B16‐OVA cells at a ratio of 100:1 compared with those from mice administrated by CryoMNs@aPD1 and CryoMNs@OVA‐DC. Collectively, these results indicate that co‐administration of OVA‐DCs and aPD1 using cryoMNs induced more potent antigen‐specific immune responses according to parameters including T cell proliferation, IFN‐γ secretion and the CTL response, compared with mono‐delivery of OVA‐DCs or aPD1 with cryoMNs.

### 2.5 Co‐delivery of OVA‐DCs and aPD1 with cryoMNs induce potent therapeutic effect

**FIGURE 5:**
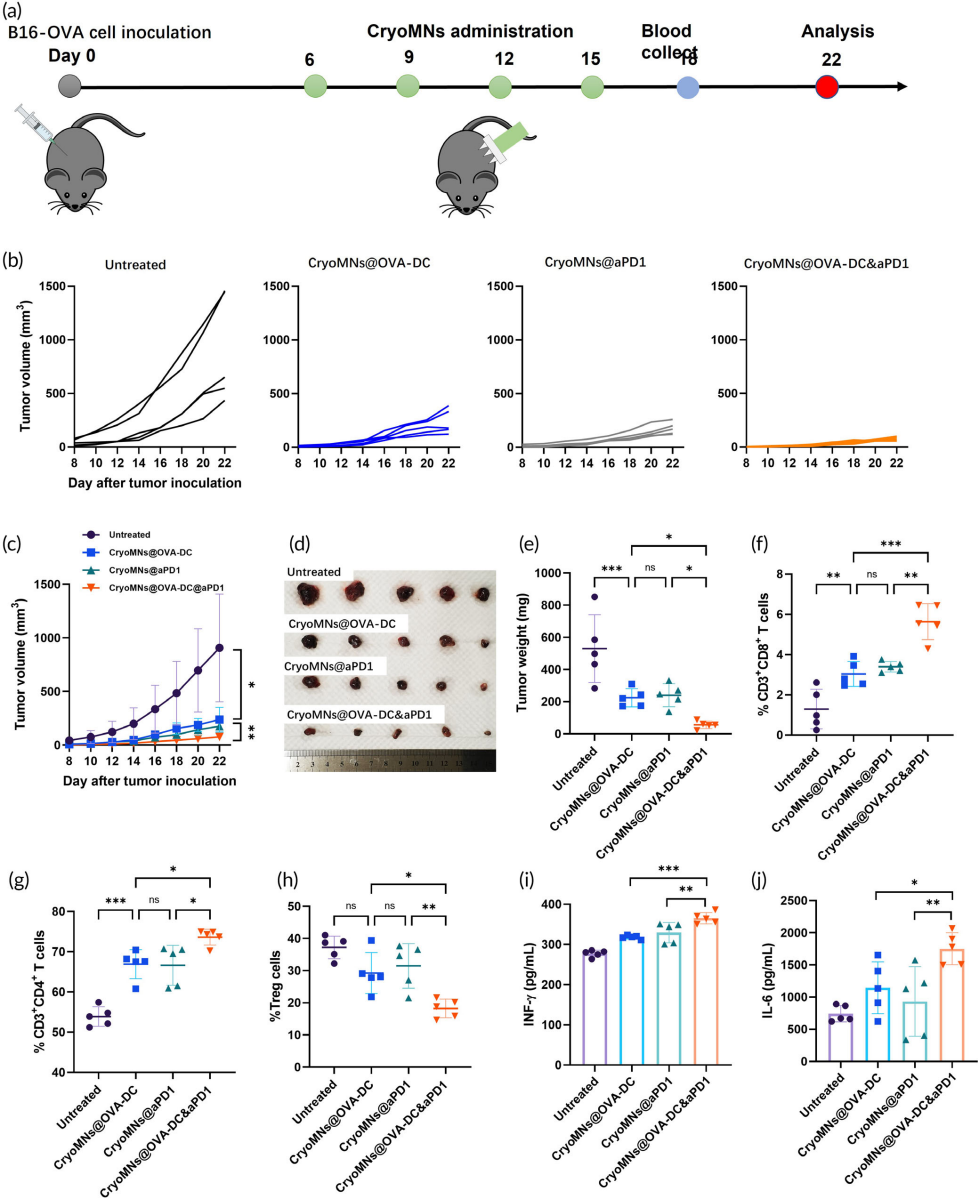
Therapeutic efficacy of administration with cryoMNs co‐encapsulating with DCs and aPD1. (a) Schematic of therapeutic administration of cryoMNs and tumor cell inoculation. (b) Individual tumor growth curves in different treatment groups. (c) Average tumor growth curve (*n* = 5). (d) Digital images of excised tumors at day 22. (e) Weight of excised tumors at day 22. Data are presented as mean ± SD (*n* = 5). Quantification of the percentage of (f) CD3^+^CD8^+^ T cells, (g) CD3^+^CD4^+^ T cells, and (h) Treg (CD4^+^ Foxp3^+^ T cells) in the tumor detected by flow cytometry. Data are presented as mean ± SD (*n* = 5). Secreted levels of (i) IFN‐γ and (j) IL‐6 in serum were collected from mice receiving various treatments on day 18. Data are presented as mean ± SD (*n* = 5). ns, *p* > 0.05, no significant difference; *p < 0.05; **p < 0.01; ***p < 0.001

Next, we studied the therapeutic effect of CryoMNs@OVA‐DCs, CryoMNs@aPD1 and CryoMNs@OVA‐DC&aPD1 against established tumors. Mice were subcutaneously inoculated with B16‐OVA melanoma cells and administrated with cryoMNs 7 days later. The detailed treatment schedule was shown in Figure 5a. There was rapid tumor growth displayed on untreated tumor‐bearing mice, while CryoMNs@OVA‐DC and CryoMNs@aPD1 were able to inhibit tumor growth (Figure 5b–d). Co‐delivery of OVA‐DC and aPD1 with cryoMNs resulted in higher efficiency in suppressing tumor growth than mono‐delivery of OVA‐DCs or aPD1. In line with the inhibition of tumor growth, the weight of tumors isolated on day 22 from the group treated with CryoMNs@OVA‐DC&aPD‐1 was also the lowest (56.2 ± 23.5 mg), compared to those from the groups treated with CryoMNs@OVA‐DC (224.4 ± 56.5 mg) and CryoMNs@aPD1 (240.6 ± 71.6 mg) (Figure 5e). Neither obvious weight loss nor evidence of toxicity or inflammation in major organs was observed in all groups during the experiment (Figures S1 and S2). Besides, the administration of cryoMNs caused no obvious hepatic damage according to the level of alanine aminotransferase (ALT) and aspartate aminotransferase (AST) **(**Figure S3). These results indicate that delivery of DC vaccines and aPD1 via cryoMNs was safe as our previous report.^21^

We also explored the immune mechanism underlying the antitumor effects was investigated. It has been reported that infiltration of effector T cells in tumor is essential for the elimination of tumor cells.^27^ Therefore, tumor‐infiltrating lymphocytes were isolated and analyzed by flow cytometry. Delivery of OVA‐DC or aPD1 with cryoMNs increased infiltration of CD8^+^ T cells and CD4^+^ T cells in the tumor, while co‐delivery of OVA‐DC and aPD1 obviously promoted these results about 1.6‐fold and 1.1‐fold, respectively **(**Figure 5f,g and Figure S4 **)**. The increase in effector T cells was correlated with the tumor suppression efficiency. Moreover, the tumor‐infiltrating CD4^+^ Foxp3^+^ T cells were analyzed. In contrast to the effector T cells, co‐delivery of OVA‐DC and aPD1 with cryoMNs significantly decreased the level of tumor‐infiltrating regulatory T cells (Tregs) (Figure 5h and Figure S4). Tregs are a specialized subpopulation of T cells that act to prevent autoimmunity by inhibiting T cell proliferation and cytokine production.^29^ Administration of CryoMNs@OVA‐DC&aPD‐1 triggered higher secretion of cytokines including IFN‐γ and IL‐6 in serum than either CryoMNs@OVA‐DC or CryoMNs@aPD1 treatment (Figure 5i,j). IFN‐γ is related to anti‐tumor efficiency, while IL‐6 is representative proinflammatory factor and related to the immune activation.^30^ Collectively, these results indicate that the co‐delivery of OVA‐DC and aPD1 using cryoMNs increased the infiltration of tumor antigen‐specific T cells in tumor tissues and upregulated the level of immunological cytokines, leading to more robust antitumor therapeutic efficacy compared with mono‐delivery of OVA‐DC or aPD1.

### 2.6 In vivo prophylactic effect of administration with cryoMNs co‐encapsulated with OVA‐DC and aPD1

**FIGURE 6:**
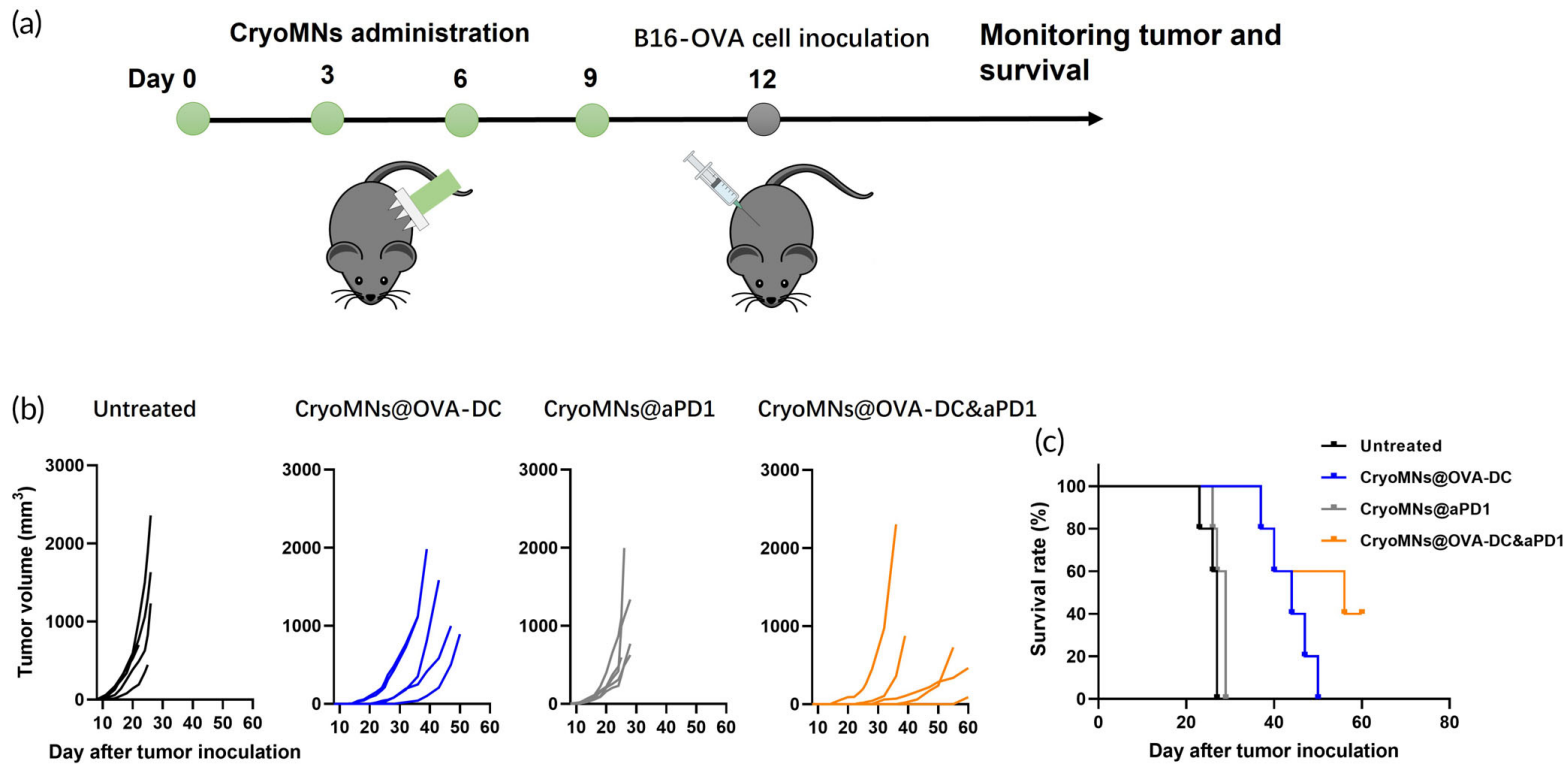
In vivo prophylactic efficacy of administration with cryoMNs co‐encapsulating with DCs and aPD1. (a) Schematic of prophylactic administration of cryoMNs and tumor cell inoculation. (b) Individual tumor growth curves of mice in different treatments (*n* = 5). (d) Kaplan–Meier survival curve of mice with different treatments after tumor challenge (*n* = 5)

We also investigated whether the co‐delivery of OVA‐DC and aPD1 using cryoMNs can act as a prophylactic vaccine in cancer immunotherapy. Although most cancer immunotherapies have focused on the use of immune therapeutics after a tumor is detected, prophylactic anti‐tumor immunotherapies can be used to prevent tumor recurrence. Mice were administrated with CryoMNs@OVA‐DC, CryoMNs@aPD1, and CryoMNs@OVA‐DC&aPD1 on day 0, 3, 6, and 9, followed by subcutaneous inoculation with B16‐OVA melanoma cells on day 12 (Figure 6a). It was found that administration with CryoMNs@aPD‐1 did not inhibit tumor growth, which was similar to the untreated group (Figure 6b). In contrast, administration with CryoMNs@OVA‐DC and CryoMNs@OVA‐DC&aPD1 greatly delayed the tumor growth compared with administration with CryoMNs@aPD1 (Figure 6b). OVA‐DC combined with aPD1 indeed improved the tumor growth inhibition efficiency, despite no significant difference. Mice without treatment and with the administration of CryoMNs@aPD1 were all dead after 30 days post tumor challenge. Co‐delivery of OVA‐DCs and aPD1 with cryoMNs was able to significantly extend the survival of mice compared to mono‐delivery of OVA‐DCs (Figure 6c). Two of five mice with administration of CryoMNs@OVA‐DC&aPD1 survived greater than 60 days post‐challenge. Taken together, these results suggest that co‐delivery of OVA‐DCs and aPD1 with cryoMNs could induce stronger tumor prevention ability compared with delivery of OVA‐DCs or aPD1 alone.

### 2.7 Discussion

This study demonstrated the feasibility of cryoMNs for packaging and co‐delivering of DC vaccines and aPD1. In the clinical settings, DC or aPD1 are usually administrated via injection with hypodermic needle.^31^, ^32^, ^33^ Injection with a hypodermic needle is always associated with obvious shortcomings, such as pain or discomfort, needle phobia, potential risk of stick injury and infection, generation of sharp wastes and the need of experienced health care provider. In contrast, DC and aPD1 delivered by cryoMNs is minimally invasive, has no biohazardous sharp wastes and can be used by less‐trained personnel. Living DC based immunotherapy generally involves frequent administration, which necessitates repeated blood collections and generation of DC vaccines. These laboursome processes are time‐consuming and costly with batch‐to‐batch variation.^34^ By using the cryoMNs technology, DCs can be generated from one‐time blood collection and packaged in cryoMNs as ready‐to‐use aliquots, which would greatly simplify the DC based immunotherapy. Intradermal administration of DC vaccines was considered superior to other administration routes, with stronger immune responses.^35^ However, intradermal injection is often performed by the Mantoux technique, which is technically challenging.^25^ Due to the special length of MNs, intradermal delivery can be easily performed with cryoMNs.^36^ Our previous study also demonstrated that intradermal administration of DC vaccines with cryoMNs induced stronger antitumorigenic ability compared with administration with subcutaneous and intravenous injections.^21^ It is worth noting that aPD1 was also administrated with cryoMNs via intradermal route, differing from other studies that intratumorally administrated aPD1.^37^, ^38^ A recent study by Francis et al. demonstrated that intradermal routes of administration of ICB monoclonal antibody (mAb) resulted in higher mAb accumulation within both the tumor microenvironment and its dLNs. This local administration route resulted in potent T cell responses.^39^ This can be used to explain that intradermal co‐delivery of OVA‐DCs and aPD1 with cryoMNs was able to achieve synergistic therapeutic effects in the inhibition of tumor growth and prevention of tumor formation in the context of the therapeutic and prophylactic setting. Our future study will comprehensively study the influences of administration routes of DC vaccines and aPD1 on the therapeutic efficacy of combinational immunotherapy by comparing cryoMNs with conventional administration routes (e.g., subcutaneous, intravenous and intratumoral injection), as well as the potential mechanism behinds these differences.

Despite these advantages of cryoMNs, there are still some translational challenges for this technology. First, since cryoMNs will melt after being removed from their storage conditions, the stability issue of cryoMNs needs to be taken into account during application. Despite integrating of cryoMNs with a handle in this study, the development of a special applicator will facilitate their easy and rapid penetration. Second, sterilization of cryoMNs is important for avoiding potential infection. In this regard, cryoMNs can be manufactured under aseptic condition. However, new sterilization methods that can be done under low temperatures are worthy of exploration for improvement of cost‐effectiveness. Third, ultracold shipping and storage conditions are required during storage conditions and transportation of cryoMNs. For example, traditional cryogenic equipment for cell storage and transportation such as −80°C refrigerators and liquid nitrogen containers, can be used for cryoMNs.

## 3 CONCLUSIONS

In summary, this study demonstrated the applicability of cryoMNs as a co‐delivery system of DC vaccines and aPD1 for combinational immunotherapy. By combination with a homemade handle, cryoMNs could be easily used during administration. CryoMNs could penetrate the porcine skin and mouse skin, successfully releasing loaded DCs and aPD1 inside skin tissue. Co‐delivery of OVA‐DCs and aPD1 with cryoMNs could induce potent antigen‐specific immune responses superior to mono‐delivery of OVA‐DCs or aPD1. Moreover, administration of cryoMNs coloaded with OVA‐DCs and aPD1 notably increased intratumoral infiltration of effector T cells and decreased the Treg cells, resulting in stronger antitumor effects in both therapeutic and prophylactic melanoma model compared with administration of cryoMNs loaded with OVA‐DCs or aPD1. These results show the great potential of cryoMNs to serve as a promising local co‐delivery platform to deliver therapeutic living cells and biomacromolecules simultaneously, not only for cancer immunotherapy, but also for various diseases where combination therapy is required.

## 4 EXPERIMENTAL SECTION

### 4.1 Materials

Sylgard® 184 silicone elastomer kit was purchased from Dow Corning (USA). Dimethyl sulfoxide (DMSO), phosphate buffered saline (PBS), sucrose, bovine serum albumin (BSA), cell strainer, and ovalbumin (from chicken egg white, OVA) were purchased from Sigma‐Aldrich (USA). DMEM, RPMI‐1640, fetal bovine serum (FBS), 10,000 U/ml penicillin–streptomycin (P/S), trypsin–EDTA, alamarBlue™ Cell Viability Reagent, LIVE/DEAD™ viability/cytotoxicity kit, 2‐Mercaptoethanol, MEM non‐essential amino acids solution, sodium pyruvate, HEPES, ACK Lysing Buffer, anti‐mouse MHC Class II (APC, clone AF6‐120.1) and CD86 antibody (APC, clone GL1), CyQUANT™ LDH Cytotoxicity Assay were purchased from ThermoFisher Scientific (USA). Mouse IFN‐γ ELISA Kit was purchased from the StemCell Pte. Ltd. (Singapore). Recombinant murine granulocyte‐macrophage colony stimulating factor (GM‐CSF) was purchased from R&D Systems (USA). Recombinant murine IL‐4 was purchased from Peprotech (USA). H&E Stain Kit (Hematoxylin and Eosin, ab245880) was purchased from Abcam (USA). Anti‐mouse CD8a (FITC, clone 53–6.7), CD4 (APC, clone RM4‐5), CD279 (aPD‐1) (Ultra‐LEAF™ Purified, clone RMP1‐14), CD279 (FITC, clone 29F.1A12), FOXP3 (PE, clone MF‐14), CD3 (PE, clone 17A2) and CD11c (FITC, clone N418) antibodies were purchased from BioLegend (USA). Mouse ELISA kit of IL‐6 and IFN‐𝛾 were purchased from Neo‐Bioscience (China). ALT and AST Activity Assay kit were purchased from Solarbio (China).

### 4.2 Animals and cells

C57BL/6 mice (Female, 6–8 weeks) were purchased from Laboratory Animal Research Unit (LARU). Animal experiments were performed in accordance with ethical approval by Animal Research Ethics Sub‐Committee of City University of Hong Kong, under the Internal Ref: A‐0493 and Animal Research Ethics Committee of Institute of Basic Medicine and Cancer (IBMC), Chinese Academy of Sciences. All mice used in the present study were housed at 20 ~ 24°C and 30–70% humidity. The light/dark cycle of the holding room was 12 h/12 h from 8:00 a.m. to 8:00 p.m. The B16 melanoma cell line transfected with ovalbumin (B16‐OVA) was gifted by the lab of Professor Jiandong Huang from School of Biomedical Sciences, The University of Hong Kong, Hong Kong. B16‐OVA cells were cultured in DMEM supplemented with 10% FBS and 1% P/S in the culture dish at 37°C and 5% CO_2_. The culture medium was changed every 2 or 3 days and culture dishes with 80 ~ 90% confluence were used for further cell experiments.

### 4.3 Generation of antigen (OVA) pulsed dendritic cells

Bone marrow‐derived DCs were isolated and generated from femur bones of C57BL/6 mice according to our previous study.^21^ Briefly, femur bones were removed from the mouse and the bone marrow was flushed by a 32G needle. After passing through the cell strainer (70 μm), marrow cells were then collected by centrifugation at 1200 rpm for 5 min. A total of 5 ml ACK lysing buffer was added to lyse blood cells, and the remaining cells were collected by centrifugation at 1200 rpm for 5 min. Cells were seeded into six‐well plate at the concentration of 2 × 10^6^ cells/ml and cultured in RPMI 1640 supplemented with 10% FBS, 2.05 mmol/L L‐glutamine, 0.1 nmol/L non‐essential amino acids solution, 1 mmol/L sodium pyruvate, 1 mmol/L HEPES, 1% P/S, 20 ng/ml of GM‐CSF, 10 ng/ml of IL‐4 and 0.056 mmol/L 2‐Mercaptoethanol. The culture medium was changed every 2 days. On the 7th day, all the non‐adherent cells (DCs) were collected. To prepare the OVA pulsed DCs (OVA‐DCs), DCs were incubated with 50 μg/ml OVA for 24 h and then collected by centrifugation.

### 4.4 Fabrication of DCs and aPD‐1 co‐encapsulated cryoMN patch

PDMS MN mold was obtained by replication of a designed metal MN template (10 × 10 array, 450 μm base diameter, 900 μm pitch, and 1200 μm height) then treated with O_2_ plasma (PDC‐36G, Kejing Materials Tech. CO. LTD.) and sterilized by 20 min UV exposure. In this study, PBS supplemented with 2% (v/v) DMSO and 100 mM sucrose was used as the cryogenic medium according to our previous study.^21^ 50 μl cryogenic medium supplemented with 1 μg/μl aPD1 was cast into mold and centrifuged at 3000 rpm for 3 min. Excess medium was collected from the substrate cavity. Then, 100 μl cryogenic medium with 1 × 10^5^ OVA‐DCs was cast into the same mold and centrifuged at 500 rpm for 1 min to allow the cells to fill up the cavities of MNs. A home‐made handle fabricated by 3D printing was vertically placed into the medium in the substrate cavity. The mold was left in 4°C refrigerator for 20 min and then transferred to −20°C for 4 h and −80°C overnight. CryoMN patch was obtained after demoulding. CryoMNs were either stored under −80°C or liquid nitrogen until use. According to the calculation, the volume of the MN cavity was about 6 μl and the total dosage of aPD1 was 6 μg per patch.

### 4.5 Characterization of cryoMN patch

The cryoMN patch was immediately imaged by a stereomicroscope (T2‐3M80, Aosvi) after being removed from the liquid nitrogen. To visualize the encapsulated DC and aPD1 inside the cryoMNs, DC stained with NucBlue™ Live ReadyProbes™ Reagent (Hoechst 33342, Invitrogen) and FITC conjugated aPD1 (FITC‐aPD1) were loaded into cryoMNs during the fabrication. Then the cryoMNs were inserted into a 1.4 wt % agarose hydrogel through a layer of parafilm. The holes created by cryoMNs in agarose hydrogel were visualized and imaged by an inverted microscope (CKX53, Olympus).

### 4.6 Skin insertion of cryoMN patch

CryoMNs loaded with stained DC and FITC‐aPD1 were inserted into porcine skin (fresh porcine cadaver skin was purchased from the local supermarket) or mouse dorsal skin by the press after being taken out of liquid nitrogen. Before MN application, the mouse dorsal hair was carefully removed. The MN‐treated skin tissue was harvested and further for the frozen section with a cryostat (NX70, Thermo Scientific). The porcine skin samples were stained with hematoxylin and eosin (H&E) and imaged by an inverted microscope (CKX53, Olympus). The mouse skin samples were directly imaged by the same microscope to identify the stained DC and FITC‐aPD1 delivered into the skin with cryoMNs.

### 4.7 In vivo immune responses after administration of cryoMNs

C57BL/6 mice were randomly divided into five groups (*n* = 5): untreated, cryoMNs, cryoMNs@DC, cryoMNs@aPD‐1, and cryoMNs@DC&aPD‐1. Each of the mice from treatment groups received administration with 4 patches of cryoMNs on day 0, 3, 6, and 9. Draining lymph nodes (dLNs) and spleen were harvested 3 days after the last MN administration and processed into a single cell suspension. To evaluate DC activation and maturation, cells extracted from dLNs were stained with anti‐CD11c, anti‐CD86 and anti‐MHCII antibodies. The stained cells were measured by a flow cytometer (BD Biosciences) and were analyzed by FlowJo software (TreeStar, version 10.0.7r2).

To evaluate the antigen‐specific T cell immune responses, splenocytes (5 × 10^5^ per well) were seeded in the 96‐well plate and re‐stimulated with 50 μg/ml OVA for 2 days. Splenocytes proliferation was evaluated using an alamarBlue cell viability assay according to the protocol provided by the manufacturer. The production of IFN‐γ in culture supernatants was measured by the mouse IFN‐γ ELISA Kit. The CTL assay was conducted following the manufacturer's protocol (CyQUANT™ LDH Cytotoxicity Assay Kit). Splenocytes (effector cells) and B16‐OVA (target cells) were then co‐cultured in 96‐well plates with the cell number ratios of 100:1. After incubation for 2 h at 37°C, the lysed target cells were quantified.

### 4.8 In vivo antitumor effect

To measure the therapeutic efficacy, 2 × 10^5^ B16‐OVA cells were inoculated subcutaneously into the right flanks of mice. After 6 days, the melanoma‐bearing mice were randomly divided into four groups (*n* = 5): untreated, cryoMNs@DCs, cryoMNs@aPD‐1 and cryoMNs@DCs&aPD‐1. Each of the mice from cryoMNs‐treated groups received administration with 4 patches of cryoMNs on day 6, 9, 12, and 15. Once the tumor became palpable, the tumor size and weight of tumor‐bearing mice were measured every 2 days. Tumor volume was calculated according to the following formula: volumes = width^2^ × length × 0.5. On day 18, serums of mice were were collected from each group of mice. The levels of IFN‐𝛾 and IL‐6 were analyzed using an ELISA kit according to the manufacturer's instructions. On day 22, the tumors were harvested for taking images by mobile photo. To evaluate the infiltration of T cells, the excised tumors were processed into a single cell suspension and stained with anti‐CD3, anti‐CD8a, anti‐CD4, and anti‐Foxp3 antibodies. The stained cells were measured by a flow cytometer (BD Biosciences) and were analyzed by FlowJo software (TreeStar, version 10.0.7r2). To evaluate the prophylactic efficacy, mice received administration of cryoMNs on day 0, 3, 6, and 9. On day 12, 2 × 10^5^ B16‐OVA cells were inoculated subcutaneously into the right flanks of mice. Once the tumor became palpable, the tumor volume was measured every 2 days.

### 4.9 Statistical analysis

Quantitative data are represented as means ± standard deviations (SD). Statistical analysis was performed using Student's *t*‐test or one‐way analysis of variance (ANOVA). Probability (*p*) values less than 0.05 were considered statistically significant.

## AUTHOR CONTRIBUTIONS

**Hao Chang:** Conceptualization (lead); formal analysis (lead); funding acquisition (equal); investigation (lead); methodology (lead); project administration (equal); resources (equal); supervision (equal); validation (lead); visualization (lead); writing – original draft (lead); writing – review and editing (equal). **Xueyu Wen:** Formal analysis (supporting); investigation (supporting); validation (supporting); visualization (supporting); writing – review and editing (supporting). **Zhiming Li:** Investigation (supporting); visualization (supporting); writing – original draft (supporting); writing – review and editing (supporting). **Zhixin Ling:** Investigation (supporting); methodology (supporting); writing – review and editing (supporting). **Yanting Zheng:** Investigation (supporting); validation (supporting); writing – review and editing (supporting). **Chenjie Xu:** Conceptualization (equal); Funding acquisition (equal); Methodology (supporting); Project administration (equal); Resources (equal); Supervision (equal); Writing – original draft (supporting); Writing – review & editing (supporting).

## CONFLICT OF INTEREST

Hao Chang and Chenjie Xu are inventors in a patent application that has been filed based on the data in this manuscript. Hao Chang is the scientific founder of Medcraft Biotech. Inc.

## Supporting information


**Figure S1.** Body weight of mice from different treatment groups post tumor inoculation in the therapeutic melanoma model.
**Figure S2.** Systemic toxicity of vaccination in different groups, including Untreated, CryoMNs@OVA‐DC, CryoMN@aPD‐1, and CryoMNs@OVA‐DC&aPD‐1. H&E analysis for mouse major organs after vaccination. Scale bar, 250 μm
**Figure S3.** Level of alanine aminotransferase (ALT) (A) and aspartate aminotransferase (AST) (B) in livers of mice in different treatment groups. Data are presented as mean ± SD (*n* = 5). ns, *p* > 0.05, no significant difference.
**Figure S4.** Representative plots of CD3^+^CD8^+^ T cells, CD3^+^CD4^+^ T cells, and Treg (CD4^+^ Foxp3^+^ T cells) in the tumor detected by flow cytometry.

## Data Availability

The main data supporting the results in this study are available within the paper and its supporting information. Additional data related to this work are available for research purposes from the corresponding author on reasonable request.
