# Supplementary material for: Co‐delivery of dendritic cell vaccine and anti‐PD‐1 antibody with cryomicroneedles for combinational immunotherapy
Source: Bioeng Transl Med. 2022 Nov 27;8(5):e10457. doi: 10.1002/btm2.10457 (PMC10487323; doi:10.1002/btm2.10457)
Supplement: Supplementary file 1 — Figure S1. Body weight of mice from different treatment groups post tumor inoculation in the therapeutic melanoma model. Figure S2. Systemic toxicity of vaccination in different groups, including Untreated, CryoMNs@OVA‐DC, CryoMN@aPD‐1, and CryoMNs@OVA‐DC&aPD‐1. H&E analysis for mouse major organs after vaccination. Scale bar, 250 μm Figure S3. Level of alanine aminotransferase (ALT) (A) and aspartate aminotransferase (AST) (B) in livers of mice in different treatment groups. Data are presented as mean ± SD (n = 5). ns, p > 0.05, no significant difference. Figure S4. Representative plots of CD3+CD8+ T cells, CD3+CD4+ T cells, and Treg (CD4+ Foxp3+ T cells) in the tumor detected by flow cytometry. [file BTM2-8-e10457-s001.docx]

**Supporting Information**

Co-delivery of dendritic cell vaccine and anti-PD-1 antibody with cryomicroneedles for combinational immunotherapy

Hao Chang^1, 2,^ *, Xueyu Wen^1^, Zhiming Li^1^, Zhixin Ling^1^, Yanting Zheng^1,3^, and Chenjie Xu ^2,^ *

^1^ The Cancer Hospital of the University of Chinese Academy of Sciences (Zhejiang Cancer Hospital), Institute of Basic Medicine and Cancer (IBMC), Chinese Academy of Sciences, Hangzhou, Zhejiang 310022, China

^2^ Department of Biomedical Engineering, City University of Hong Kong, Hong Kong SAR, China

^3^ College of Pharmaceutical Science, Zhejiang University of Technology, Hangzhou 310014, Zhejiang, China

E-mail: [changhao@ibmc.ac.cn](mailto:changhao@ibmc.ac.cn); chenjie.xu@cityu.edu.hk

**Figure S1**. Body weight of mice from different treatment groups post tumor inoculation in the therapeutic melanoma model.


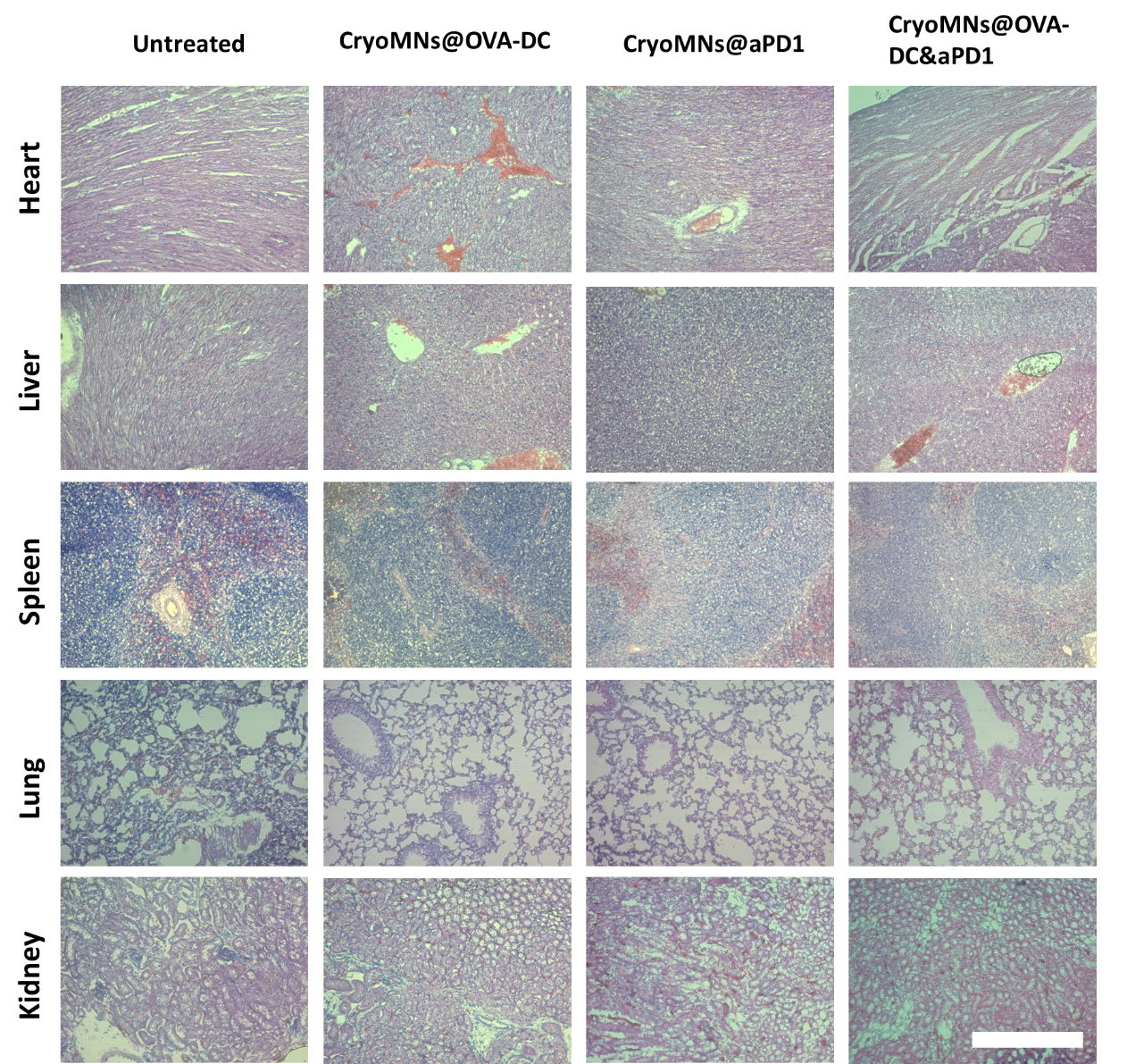


**Figure S2.** Systemic toxicity of vaccination in different groups, including Untreated, CryoMNs@OVA-DC, CryoMN@aPD-1, and CryoMNs@OVA-DC&aPD-1. H&E analysis for mouse major organs after vaccination. Scale bar, 250 μm


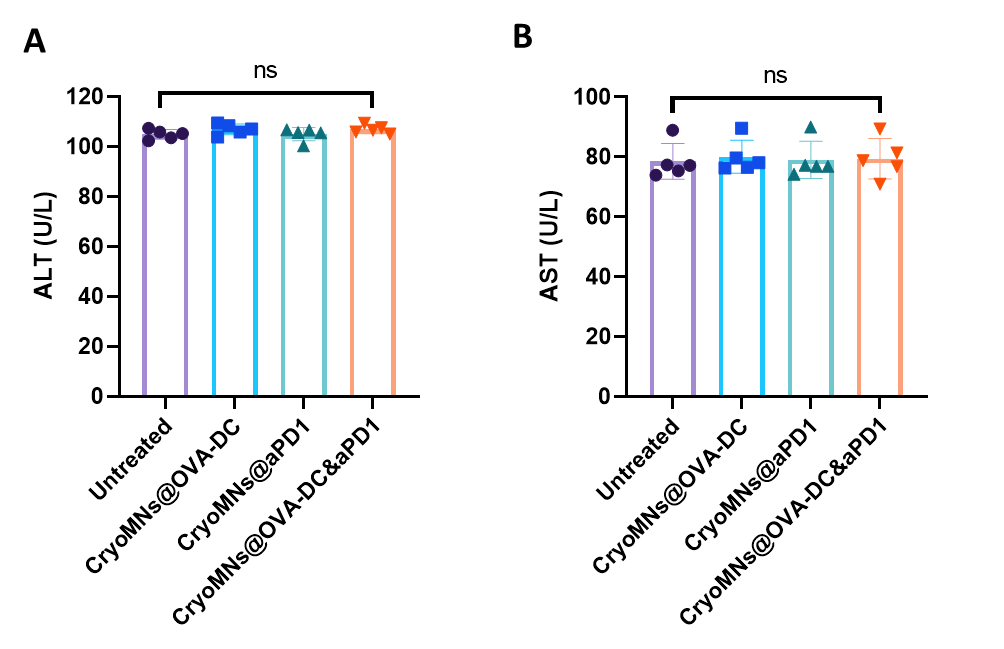


**Figure S3.** Level of alanine aminotransferase (ALT) (A) and aspartate aminotransferase (AST) (B) in livers of mice in different treatment groups. Data are presented as mean ± s.d. (n = 5). ns, P > 0.05, no significant difference.


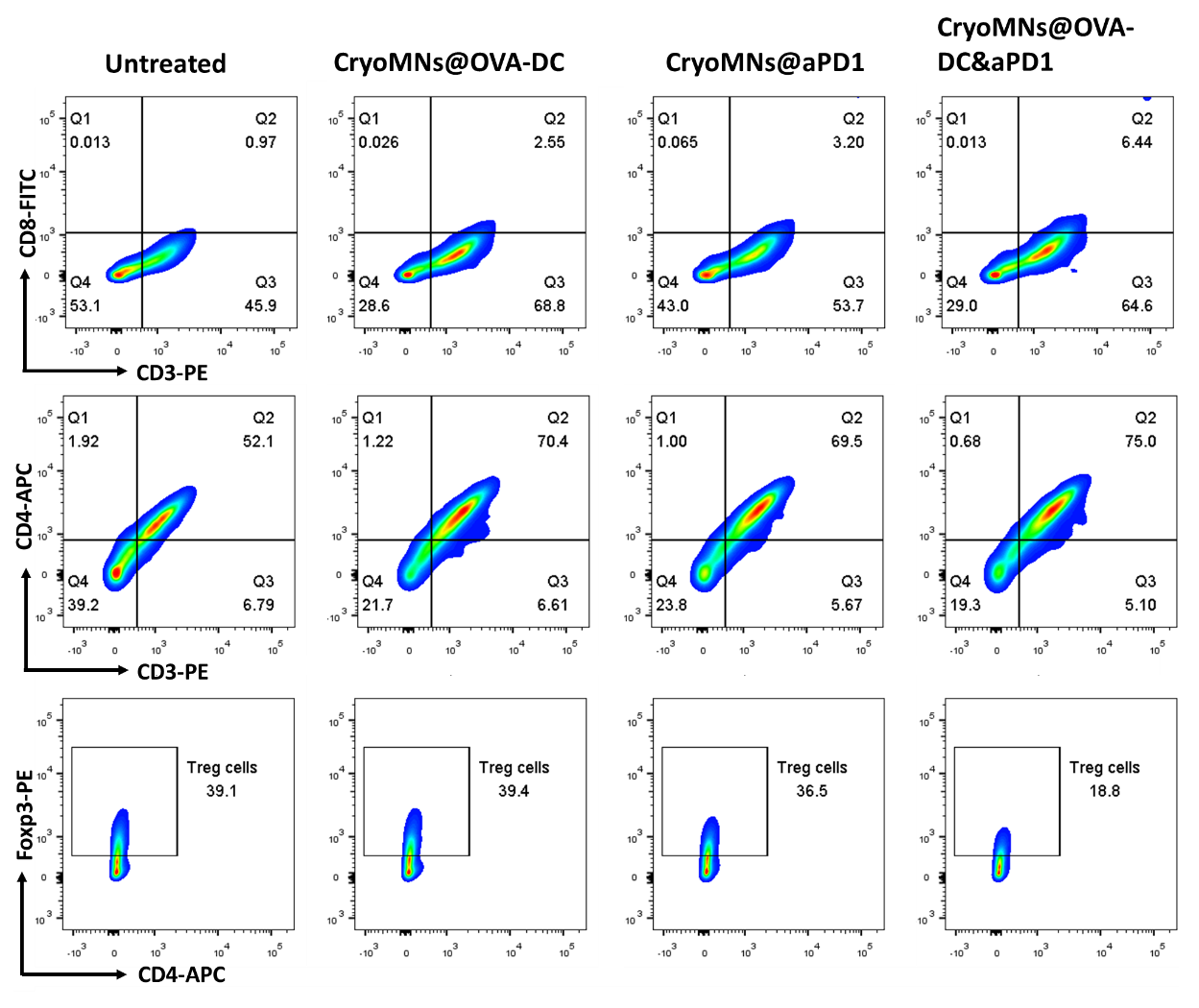


**Figure S4.** Representative plots of CD3^+^CD8^+^ T cells, CD3^+^CD4^+^ T cells, and Treg (CD4^+^ Foxp3^+^ T cells) in the tumor detected by flow cytometry.
